# ORdensity: user-friendly R package to identify differentially expressed genes

**DOI:** 10.1186/s12859-020-3463-4

**Published:** 2020-04-07

**Authors:** José María Martínez-Otzeta, Itziar Irigoien, Basilio Sierra, Concepción Arenas

**Affiliations:** 1 Department of Computation Science and Artificial Intelligence, University of the Basque Country UPV/EHU, Donostia, Spain; 2 Department of Computation Science and Artificial Intelligence, University of the Basque Country UPV/EHU, Donostia, Spain; 3 Department of Computation Science and Artificial Intelligence, University of the Basque Country UPV/EHU, Donostia, Spain; 4 Department of Genetics, Microbiology and Statistics, University of Barcelona, Barcelona, Spain

**Keywords:** Differentially expressed gene, Multivariate statistics, Outlier, Parallel implementation, Quantile, R package

## Abstract

**Background:**

Microarray technology provides the expression level of many genes. Nowadays, an important issue is to select a small number of informative differentially expressed genes that provide biological knowledge and may be key elements for a disease. With the increasing volume of data generated by modern biomedical studies, software is required for effective identification of differentially expressed genes. Here, we describe an R package, called ORdensity, that implements a recent methodology (Irigoien and Arenas, 2018) developed in order to identify differentially expressed genes. The benefits of parallel implementation are discussed.

**Results:**

ORdensity gives the user the list of genes identified as differentially expressed genes in an easy and comprehensible way. The experimentation carried out in an off-the-self computer with the parallel execution enabled shows an improvement in run-time. This implementation may also lead to an important use of memory load. Results previously obtained with simulated and real data indicated that the procedure implemented in the package is robust and suitable for differentially expressed genes identification.

**Conclusions:**

The new package, ORdensity, offers a friendly and easy way to identify differentially expressed genes, which is very useful for users not familiar with programming.

**Availability:**

https://github.com/rsait/ORdensity

## Background

Analysis of gene expression using microarray or RNA-Seq technologies is a very important task and the main goal is to identify a small number of informative genes whose patterns of expression differ according to the experimental conditions. An important challenge is the discovery of these differentially expressed genes (DEGs). This is because there is a large number of genes, a relatively small number of samples and it is important to identify which genes, independent of the sample studied of the same disease, are selected as DE genes. In order to identify a list of DEGs, different procedures were introduced in the scientific community. Significance Analysis of Microarrays (SAM) [1] works with a modified t-test introducing a factor to minimize the effect of small per-gene variances. An integrated solution for analyzing data from gene expression experiments is provided by limma [2, 3], an R package for Bioconductor [4]. The empirical Bayes method (eBayes) [5] also uses moderated t-statistics, where, instead of the global or single gene estimated variances, a weighted average of the global and single-gene variances is used. A different approach, the ORdensity procedure, was recently introduced [6]. This method returns three measures which are related to the concepts of outlier and density of false positives in a neighbourhood, and this allow us to identify the DEGs with high classification accuracy. The first measure is an index called OR, previously introduced in [7, 8], that identifies outliers; the other two measures, called false positives in a neighbourhood (FP), and density of false positives in a neighbourhood (dFP) were introduced in [6]. This new procedure has been implemented in the ORdensity package described below.

Ideally, effective software making the identification of differentially expressed genes possible on desktop computers should facilitate data manipulation and should be easy to carry out. It should also offer understandable outputs to give access to a wide range of users. Furthermore, due to the large dimensionality of the data sets used it should implement fast procedures. With these objectives in mind, we developed the ORdensity software implemented as an R package and offers a friendly and easy-to-use tool to perform the ORdensity method.

## Implementation

In this section, the structure of the package and the functions implemented are explained. The ORdensity package was developed for the free statistical R environment (http://www.r-project.org) and runs under all major operating systems. We do not delve into details of the underlying statistical methodology that can be consulted in [6], and only the main results are presented.

### Background

**Fig. 1:**
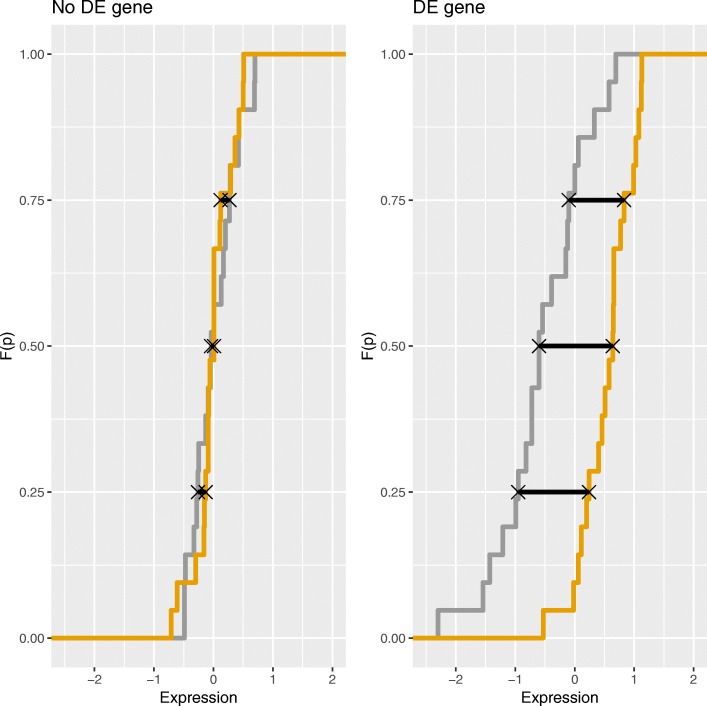
Visualization of $\hat {F}_{X_g}^{-1}(p)- \hat {F}_{Y_g}^{-1}(p)$ differences for *p*∈*C*_*p*_={0.25,0.5,0.75} for two genes. In the left side, a gene whose expressions in conditions X and Y are not differentially expressed (No DE gene); in the right side, a gene that is differentially expressed in conditions X and Y (DE gene)

Let *M* be an *n*×*s* matrix containing the expression level of *n* genes under two experimental conditions measured in *s*_1_ and *s*_2_ samples for experimental condition 1 and 2, respectively (*s*_1_+*s*_2_=*s*). Let *X*_*g*_ and *Y*_*g*_ be the random variables representing the expression level of gene *g* in conditions 1 and 2, respectively (*g*=1,...,*n*). The proposed approach focuses on the differences of quantiles between samples: $V_{gp} =F_{X_{g}}^{-1}(p)- F_{Y_{g}}^{-1}(p)$, *p*∈*C*_*p*_ where *C*_*p*_ is a set of probabilities. As we can observe in Fig. 1, a gene *g*, whose expressions under the two conditions were considered not differentially expressed (left) would verify that $F_{X_{g}}^{-1}(p) = F_{Y_{g}}^{-1}(p)$, where *F* is the cumulative distribution function, and *p*∈[0,1]. Otherwise, gene *g* is differentially expressed or it is important (right). Therefore, matrix **V**=(*v*_*gp*_) with $v_{gp} = \hat {F}_{X_{g}}^{-1}(p)- \hat {F}_{Y_{g}}^{-1}(p)$, for *g*=1,…,*G* and *p*∈*C*_*p*_ must contain small values corresponding to the majority of no DEGs. The most differentially expressed genes should show a different behaviour, hence, they can be considered as outliers in **V**. Thus, the approach attempts to find outliers in **V** which can be identified as differentially expressed genes. In a first step, given a fixed *α*∈(0,1), the procedure reduces the number of genes selecting *potential* differentially expressed genes. In a second step, the method identifies the differential expressed genes among the *potential* ones using three indexes. The index *OR*, previously introduced in [7, 8], which identifies outliers, and two measures called false positives in a *K*-Nearest Neighbourhood (FP) and density of false positives in a *K*-Nearest Neighbourhood (dFP) that are related with the false positives obtained by the permutation sampling (see [6] for more details about these indexes, *Subsections 2.1* and *2.2*). Furthermore, the procedure clusters the *potential* DE genes according to the values of these indexes. The motivation behind the clustering is to distinguish those false positive genes that score high in OR and low in mean FP and density, but are similar to simulated permuted cases and we can therefore conclude they are not genuinely DEGs.

### Input

The input data are the corresponding expression level of *n* genes under two experimental conditions measured in *s*_1_ and *s*_2_ samples. Let M1 and M2 be the two *n*×*s*_1_ and *n*×*s*_2_ matrices containing the samples for the experimental conditions 1 and 2, respectively. As the package does not include any pre-processing algorithm, if it is necessary, matrices M1 and M2 must be previously normalized or transformed in a convenient way. The aforementioned first step of the method is carried out by building the object called ORdensity.

### ORdensity object

The ORdensity object is an S4 class created to gather the *potential* differentially expressed genes given a value *α*∈(0,1). The object has the following slots:

- **Slots**
  Exp_cond_1 = ~matrix~, Exp_cond_2 = ~matrix~, the two matrices M1 and M2 (*n*×*s*_1_ and *n*×*s*_2_) containing the expression level for each gene under the two experimental conditions.
  labels, vector of characters identifying the genes, by default rownames(Exp_cond_1) is inherited. If NULL, the genes are named ‘Gene1’, …, ‘Gene*n*’ according to the order given in Exp_cond_1.
  B = ~numeric~, by default the method considers 100 permutations in order to generate values associated with genes which are not differentially expressed (see [6], Subsection *First step: finding potential differentially expressed genes*).
  scale = ~logical~, by default scale = TRUE. It is advisable to scale the differences between quantiles when the variability of genes among different types of samples is different.
  alpha = ~numeric~, by default alpha = 0.05 is the value used by the method to calculate the percentile (1−*α*)100*%* of all the elements of the matrix with the permuted samples. Only genes with *OR* value above the (1−*α*)100*%* percentile in the permuted distribution are considered as *potential* DEGs.
  fold = ~numeric~, by default fold = 10 (see [6], Subsection *Second step: identifying differentially expressed genes* and *Note 3*).
  probs = ~numeric~, by default probs = c(0.25, 0.5, 0.75) are the considered quantiles.
  weights = ~numeric~, by default weights = c(0.25, 0.5, 0.25) are the weights given to the considered quantiles in probs. It may be interesting to give greater importance to some of them, therefore different weights can be introduced.
  numneighbours = ~numeric~, number of Nearest-Neighbours to consider, by default.
  numneighbours = 10
  numclustoseek = ~numeric~, maximum number of clusters that are considered when looking for the best partition, by default numclustoseek = 10.
  parallel = ~logical~, by default, no parallelizing is enabled. To enable it, set parallel = TRUE.
  nprocs = ~numeric~, number of processes launched when the option parallel is enabled. The default value is the number of processors.
  replicable = ~logical~ and seed =~numeric~, it is also possible to enable or disable replicability, and to pass the seed to the pseudorandom number generator. The default values are replicable = TRUE, seed = 0 with the function using the given seed to set the random generator. If replicable = FALSE, no seed is used.
- **Usage**
  Following the standard procedure in R, an instance of a class ORdensity is created via the new() constructer function:
  new(~ORdensity~, Exp_cond_1 = M1, Exp_cond_2 = M2)
  Slots Exp_cond_1 and Exp_cond_2 are compulsory since they contain the expression level of genes. When a slot is not specified, the default value is considered. First, the S4 object of class ORdensity must be created. Then, the *potential* DEGs are identified and gathered in the object itself. The rest of the functions in the package are based on this object so that useful information for the user is extracted and computed.

#### Function summary

Once the *potential* DEGs genes are in the object ORdensity, it is important to find out what the main patterns among them are. That means to look for clusters of genes that have similar *OR*, *FP* and *dFP* values. The motivation of the clustering is to distinguish those false positives that score high in *OR* and low in mean *FP* and density, but are similar to other known false positives obtained by the permutation sampling. The *potential* genes are clustered using the Partition Around Medoids (PAM) method [9] based on indexes *OR*, *FP* and *dFP* once they are scaled. By default, the number of clusters is selected according to the silhouette analysis [10]. Function summary provides a general overview of the obtained clusters. It returns the number of *potential* genes in each cluster, the characteristics of the clusters in terms of the indexes *OR*, *FP* and *dFP* and also the identification labels of the genes in the cluster.

- **Usage**
  summary(object, numclusters)
- **Arguments**
  object, an object of class ORdensity.
  numclusters, optional, an integer number indicating the number of clusters the genes are partioned. By default NULL, the number of clusters is calculated according to silhouette index as mentioned before.
- **Value**
  A list of *k* lists where *k* is the best number of clusters found. The clusters are ordered based on their importance, that is, the first one is the most important and the last one is the least important, in the sense that the most differentially expressed genes will be in the first cluster, and so on. Each list has the elements:
  numberOfGenes, number of genes in the cluster.
  CharacteristicsCluster, matrix with mean values and standard deviation of variables *OR*, *FP* and *dFP* for each cluster.
  genes, identification of the genes in the cluster.

#### Function findDEgenes and function preclusteredData

We should also check a more detailed summary of the object and obtain the genes identified as DE genes. Following [6], two types of differentially expressed gene selection can be made:

**ORdensity strong selection:** take as differentially expressed genes those with a large *OR* value and with *FP* and *dFP* equal to 0.

**ORdensity relaxed selection:** take as differentially expressed genes those with a large *OR* value and with small *FP* and *dFP* values. As a reference to look for small values of *FP* and *dFP*, the expected number of false positive neighbours is computed.

Function findDEgenes gives the genes identified as DE according to the strong and relaxed selection. The genes are presented in the clusters obtained, by default, using the PAM method and the silhouette index as previously mentioned. For those users that want to study different clustering methods, function preclusteredData offers the description of all the *potential* DE genes in terms of indexes *OR*, *FP* and *dFP* in only one table. Therefore, the user can easily apply different approaches in order to discover similar gene patterns.

*2.3.2.1 ****Function**** findDEgenes*

- **Usage**
  findDEgenes(object, numclusters)
- **Arguments**
  object, an object of class ‘ORdensity’.
  numclusters, optional, an integer number indicating the number of clusters the genes are partitioned. By default NULL, the number of clusters is calculated according to the silhouette index as previously mentioned.
- **Value**
  The function returns a list with the following elements:
  neighbours, number of Nearest-Neighbours considered (inherits from slot numneighbours of the ORdensity object).
  expectedFalsePositiveNeighbours, number of False Positive Neighbours expected within the Nearest Neighbourhood under the uniform distribution that corresponds to the proportion permuted cases among all the *potential* DEGs and permuted cases (see [6], Subsection *Second step: identifying differentially expressed genes*).
  clusters, a list of data.frames. Each data.frame corresponds to a cluster gathering the indexes: *OR*, *FP*, *dFP* as well as the labels obtained by the aforementioned Strong and Relaxed selection criteria.

*2.3.2.2 ****Function**** preclusteredData*

- **Usage**
  preclusteredData(object)
- **Arguments**
  object, an object of class ‘ORdensity’.
- **Value**
  The function returns a data.frame with all *potential* DEGs, indicating which are identified as DEGs by the strong and the relaxed selection.

#### Function plot

A plot with a representation of the *potential* genes based on *OR*, *FP* and *dFP* can also be obtained using function plot.

- **Usage**
  plot(object, numberclusters)
- **Arguments**
  object, an object of class ‘ORdensity’.
  numclusters, optional, an integer number indicating the number of clusters the genes are partitioned. By default NULL, the number of clusters is calculated according to the silhouette index as previously mentioned.
- **Value**
  This function returns a plot with a representation of the *potential* genes based on *OR* (vertical axis), *FP* (horizontal axis) and *dFP* (size of the symbol is inversely proportional to its value). Moreover, genes identified as DE by the relaxed selection are represented by the symbol “ △”.

### Installation

The package is hosted in GitHub and to install the package from the repository, just run the following codelibrary(devtools)install_github(’rsait/ORdensity’)

This package requires the cluster library to be installed; otherwise it will automatically install and load it. Likewise, the parallel, foreach and doRNG libraries are used for parallelization. For the computation of the distances, the distances library is used.

To start working with the package, just load it in the R environment with the following command

library(ORdensity)

### Parallel implementation

An intrinsic computational issue in this context is the enormous computational burden that is involved. Let us take into account that to compute the median of the distances between every pair of genes in a set of *n* genes, *n*(*n*+1)/2 distances need to be calculated and stored. The running time of the fastest algorithm to find the median of a list ([11]) is *O*(*k*) on average and *O*(*k*^2^) in the worst case, where *k* is the number of elements in the list. As the number of distances is *n*^2^/2, and there are *B* permutation replicates, the average running time of the median of all the replicates is *O*(*Bn*^2^), with *O*(*Bn*^4^) in the worst case. The space requirements are of *n*^2^/2 8-byte floating point numbers to store the distances. As the implementation uses a R dist object that has to be converted into a matrix before computing the median with Rfast::med, the actual space requirement doubles. For example, in the case of the distances between 10,000 genes, an object with a size of 762.9 Mb is created in each replication. Furthermore, more temporary space allocation is done during the execution, as for example when performing subsetting over a matrix, or when computing the median. Due to R being an interpreted language, the memory management is not so efficient as in other compiled languages as C.

**Fig. 2:**
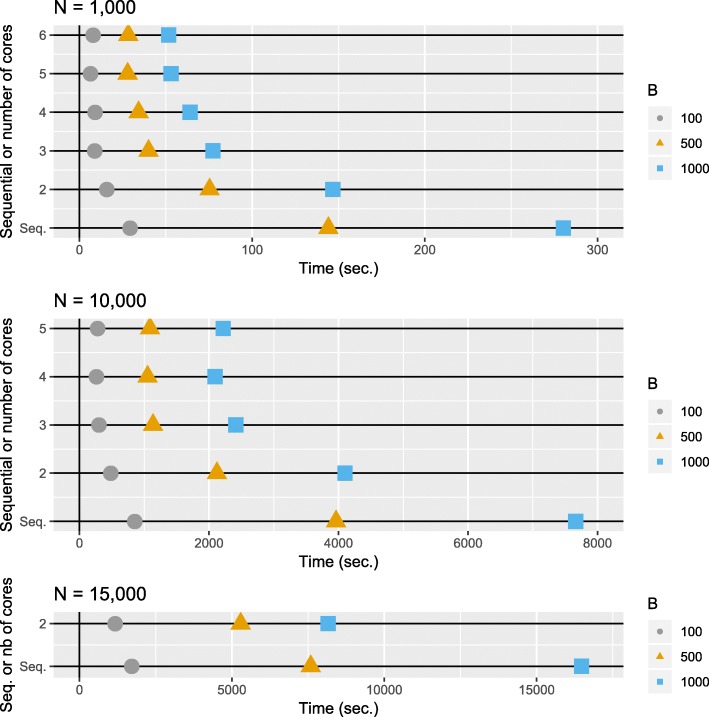
Running times for simulated data with different numbers of genes, different numbers of cores and different number of permutations used in the bootstrap procedure. In the vertical axis the type of execution: sequential or the number of processes working

We alleviated this problem by means of a parallel implementation of the bootstrap procedure. R uses a garbage collection mechanism to claim unused memory when needed, and the release of memory by individual processes when working in parallel has not been optimized. These facts make the parallel execution being sometimes slower than the sequential version due to an intensive use of swap memory. The parallel execution can be activated calling the ORdensity objects constructor with the parameter parallel = TRUE. By default, it is set to FALSE. When parallel execution is selected, the function uses the libraries parallel, foreach and doRNG. The first two are needed for parallel execution of the code, while doRNG enables replicability of pseudorandom number generations in a parallel environment. When the parallel option is enabled, the user can choose the number of processes to be run in parallel. When no number is specified, a number of processes equal to the number of processors in the machine is launched. This improves the speed, but at the cost of a higher memory load, because several objects containing the list of distances of each permutation replication will coexist in the memory at the same time. The researcher will have to take into account this trade-off, and be wary of possible memory crashes due to RAM exhaustion or of serious slowing down due to intensive use of swap memory. We conducted a series of experiments to assess the runtime. We used data sets involving different numbers of genes, from 1,000 to 15,000, and we considered different numbers of bootstrap samples, where B is equal to 100, 500 and 1,000, respectively. All the experimentation was performed on a personal computer (Intel i7-7700, 8 cores, 3.60GHz, 16 GB of RAM and 7.5 GB of swap space) running Ubuntu 16.04. Without the memory management issues previously described, based on the time complexity of the algorithm, the execution time should increase linearly with the number of bootstrap replicates, quadratically with the number of genes (estimating average time for the computation of the median), and should decrease linearly with the number of processes. In our experimental setup we observe that when working in the low range of the number of genes, processes and bootstrap replications the expected behaviour is obtained. Nevertheless, in the high end of the values of the aforementioned parameters there is a clear decrease in the performance. Even in some cases, the execution crashed due to exhaustion of RAM and swap space. For instance, when *N*=1,000, using up to 5 or 6 cores the execution times reduces roughly as expected (using 6 cores, the execution is 5.4 times faster than the sequential execution; 51.5 sec. and 280.02 sec., respectively). For *N*=10,000 genes, the improvement is observed up to 4 cores (with 4 cores the execution time is 3.7 times lower compared to the sequential implementation; 2093.1 sec. and 7662.7 sec., respectively). For big number of genes (*N*=15,000) the execution in parallel offers an improvement when only 2 cores are working being the execution in parallel 2 times faster (8157.5 sec and 16477.6 sec., respectively). When more cores were working the previously mentioned memory issues prevail. See Fig. 2 for details.

## Results and case of use

In addition to the experiments performed in our own computer, a code capsule has been created to allow the readers to experiment without having to install anything in their machines. The code capsule runs Ubuntu 18.04, and the versions of the installed software are the following: R 3.5.3, libgsl-dev 2.4+dfsg-6, Rfast 1.9.3, cluster 2.0.8, distances 0.1.7, doParallel 1.0.14, doRNG 1.7.1, foreach 1.4.4 and rngtools 1.3.1. The ORdensity package ensures reproducibility for a given configuration. In particular, the results of sequential and parallel computation could differ slightly due to the use of a different pseudorandom generator, but several sequential executions would return the same results, as well as several parallel executions even using different number of processes.

ORdensity stands out for its simplicity and ease of use. For example, consider the following simulated data, called simexpr, included in the package. We assumed a total of 1000 genes, among which 100 were generated as differentially expressed genes. The expression levels of all no DE genes were generated by *N*(0,1) distribution in both conditions 1 and 2. The DE genes were generated using the *N*(0,1) and *N*(*μ*_*g*_,1) distributions for conditions 1 and 2, respectively, with |*μ*_*g*_|=*Δ*. Parameter *Δ* sets the importance of gene *g*, where the bigger *Δ* is, the more important gene *g* is. We considered *Δ* in {1.5,2,3}.

To summarize, each row *g* in simexpr corresponds to a simulated gene. The first column indicates whether gene *g* is DE or not. The second column contains *Δ* values. Columns 3-32 and 33-62 have the expression levels under experimental condition 1 and 2, respectively.

First, we extract the samples from each experimental condition from the simexpr database, and the created S4 object which was stored in myORdensity.


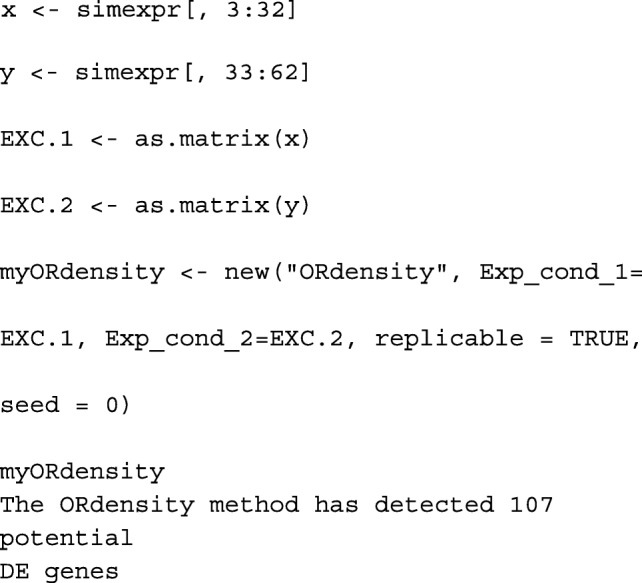


Thus far, the first step of the procedure is performed, and it detected 107 genes as *potential* DEGs that are stored. The identified *potential* genes turned out to be clustered in two clusters:


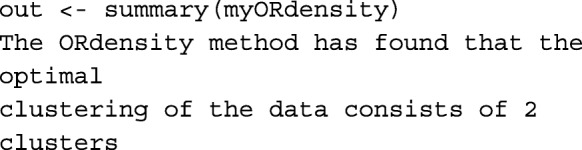


We applied the summary function without specifying the number of clusters the genes are partitioned and we got a message informing that the best number of clusters is 2. As a result, a description of the clusters is calculated. For instance,


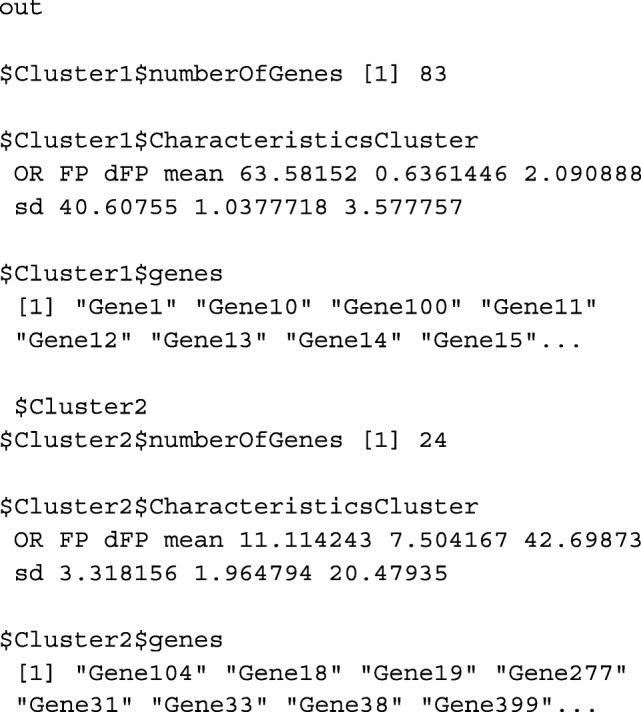


Thus, the procedure considers 107 genes as *potential* DEGs and clustered them in two clusters with 83 and 24 genes respectively. As the clusters are ordered in decreasing order according to the value of the mean of the OR statistic, we can see that the mean is higher in the first cluster (63.58) than in the second one (11.11), which means that the first group is made up of genes whose expression is very different in the two groups. The second is composed of genes with expressions that do not differ so much between groups.

**Fig. 3:**
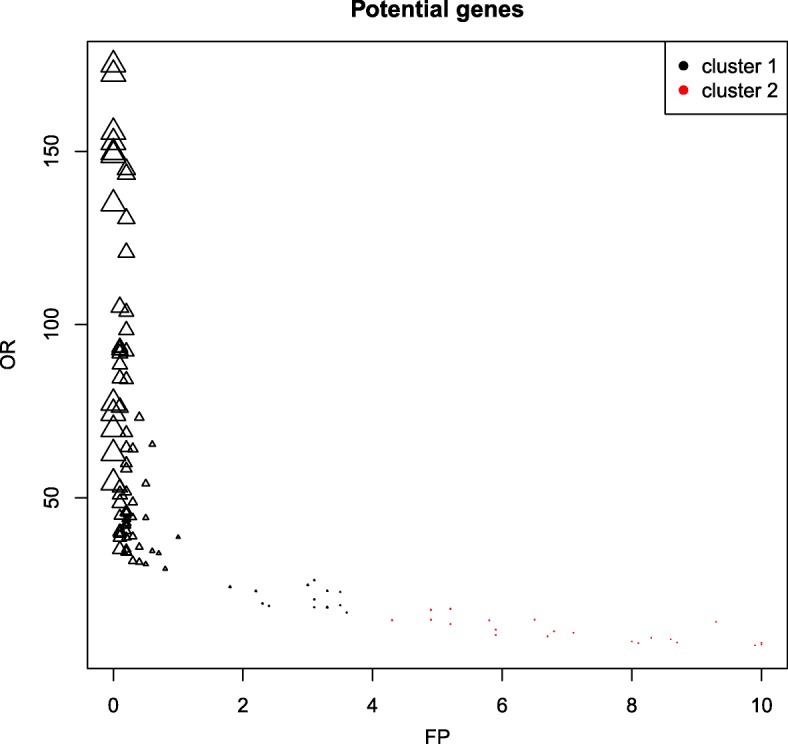
For the simexpr data set, representation of the potential genes based on *OR* (vertical axis), *FP* (horizontal axis) and *dFP* (size of the circle is inversely proportional to its value). Genes identified by the relaxed selection as DEGs are indicated by the symbol “ △”; in red and blue, genes belonging to cluster 1 and cluster 2, respectively

Using plot(myORdensity) the user can visualize the clusters. Figure 3 shows the values of indexes *OR*, *FP*, and *dFP* for the *potential* selected genes. In the vertical and horizontal axes, values *OR* and *FP*, are presented respectively. The size of the symbols is inversely proportional to *dFP*. As we can observe, the first cluster has genes with very high *OR* values as well as low *FP* and *dFP* values. Therefore, as we have pointed out, this cluster contains, as expected, the most differentially expressed genes.

In order to determine which genes are considered as DEGs, we calculated more detailed clustering output with,


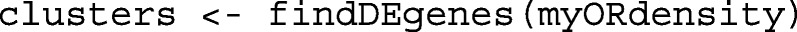


The information is shown in slots, along with the obtained partition of genes:


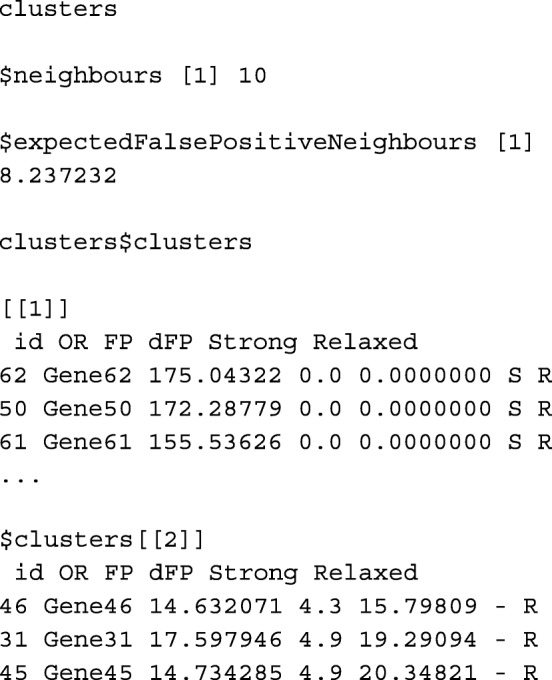


With this data, the package gives the following information, $expectedFalsePositiveNeighbours is 8.24. Thus, for a gene and according to a uniform distribution, on average, the expected number of false positives among the 10-nearest neighbours is 8.24, because considering all data, the proportion of permuted samples is 82.37%. As this is the threshold used for the relaxed selection, with this selection the procedure identifies 97 DEGs, where 12 of them were also selected by the strong selection.

As we can observe in Table 1, 83 of these 97 genes identified as DEGs are in cluster 1 (*#*cluster 1 = 83) and 14 of them are in cluster 2 (*#*cluster 2 = 24). In this simulated data, we know by construction which genes were generated as DEGs and it is easy to check that 96 of the 97 genes identified as DEGs by the relaxed selection are truly DEGs, so, we only find one false positive. The 4 genes simulated as DEGs but not selected by the procedure (false negatives) were associated to the smallest value *Δ*=1.5, one of them was not considered as *potential* DE gene and the other 3 were considered *potential* genes.

**Table 1:** Using the simexpr data set, distribution among the two clusters of the genes identified by ORdensity as DEGs

*n*	*Δ*	Identified DEGs	
True DE genes		Cluster 1	Cluster2
35	3	34 (9S/25R)	1 (0S/1R)
32	2	31 (2S/29R)	0
33	1.5	18 (1S/17R)	12 (0S/12R)
Not true DE			
900	-	0	1 (0S/1R)

In brackets the number of DEGs selected by strong (S) and relaxed (R) criterion, respectively

In order to evaluate the impact in the results of different initial seeds (in sequential and parallel executions the random seeds are handled differently), we repeated 100 times, with different inital seeds, and analyzed the results on the simexpr database. The mean number of genes detected as *potential* DE genes was 106.94 with 0.62 as standard deviation, and 891 genes never were considered *potential* DE genes. Regarding the 100 DE genes, one of them never was considered as *potential* DE gene, while the other 99 were always included in the set of *potential* DE genes. Between these 99 genes, 2 never were identified as DE genes; 1 was only detected as DE gene in less than 25 runs, and the other 96 genes were always identified as DE genes. The assignation of the label “strong” leads to a bigger variability because in fact it is a discretization of a quantitative variable (FP) given a threshold value at 0. Nevertheless, the variability of each DE gene concerning his FP values among the 100 repetitions is low: the minimum value for the standard deviation was 0.00 and the maximum 0.67, for variable FP that ranges between 0 and 10.

## Discussion

As we have shown, our ORdensity package requires minimal user intervention and obtains results in only a few instructions. The user can identify DEGs with data sets containing large number of genes (<20,000). When the user runs ORdensity can get, in addition to the results, one graph which shows the values of the three indexes used for the DEGs identification. Furthermore, ORdensity allows the user to modify the weights of the quantiles if some of them are considered more important than the others. Users can also change the cluster method and the number of clusters that, by default, are calculated using PAM procedure and the silhouette criterion. Because to guarantee reproducible analysis the method uses permutation sampling, the user can fix the seed to set the random generation. Moreover, to analyze a large number of genes and to have small run-times, parallelization across the permutation sampling procedure was implemented. However, one limitation of the method is the long execution time when the number of genes is large (>10000), an issue that is only partially improved with the parallelization, although it alleviates this inconvenience. Furthermore, like many permutation-based methods, the program requires large amount of memory to store intermediate data and the replicates of the original data matrix. Although this fact may limit the use of the program, ORdensity should be understood as an alternative program to the existing ones. Since its approach is innovative and different to other methods, it can shed light to identify interesting genes that would not be detected with other techniques. Moreover, the method for identifying DE genes implemented in the package, as it was pointed out in [6], avoids some of the shortcomings of the individual gene identification and it is stable when the original sample is changed by subsamples.

As future work, we intend to improve the running time and needed memory space by two different ways. The first one is to develop a version that makes use of Nvidia GPUs for the computation of distance matrices, and the second one is to test median approximations not so demanding computationally. GPU computation could lower the computation time when building the distance matrix, but it would still be needed to store the whole matrix in order to find the median. On the other hand, the remedian [12] algorithm (median of medians) could reduce drastically the needed memory, but at the expense of returning only an approximation to the median. Further experiments would need to be conducted to test if the ORdensity results are similar using the median or the remedian.

Although due to sequential and parallel computation, the use of a different pseu-dorandom generator may slightly affect the results, the biological impact that it can have is small, since the results vary very slightly and only affect those very poorly expressed genes.

Finally, it is worth to remark that ORdensity allows practitioners to perform their applied research in a user-friendly environment.

## Conclusions

ORdensity is a free and comprehensible R package available to the biomedical community. This computational tool is designed to identify DEGs following the method introduced in [6]. In few sentences, this tool executes an efficient and accurate analysis producing a list of differentially expressed genes, it requires a minimal user expertise and it displays the results in an easy way to interpret them. All these features make ORdensity powerful software for studies of DEGs.

## Availability and requirements

Project name: ORdensity Project home page: https://github.com/rsait/ORdensityOperating system(s): Platform independent Programming language: R 3.5.3 or higher Other requirements: Rfast 1.9.3 or higher, cluster 2.0.8 or higher, distances 0.1.7 or higher, doParallel 1.0.14 or higher, doRNG 1.7.1 or higher, foreach 1.4.4 or higher, rngtools 1.3.1 or higher License: GNU GPLv2+ Any restrictions to use by non-academics: no restrictions

## Data Availability

The only data set used is the simulated data set simexpr that is included in the package.

## Abbreviations

DE: Differentially Expressed
DEGs: Differentially Expressed Genes
*dFP*: Average density of false positive permuted cases in k-NN
*FP*: Average number of false positive permuted cases in k-NN
limma: Linear models with Empirical Bayes statistic
*OR*: Outlier Robust index
PAM: Partition Around Medoids clustering method
SAM: The Significant Analysis of Microarrays
